# Assessing the function of pneumococcal neuraminidases NanA, NanB and NanC in *in vitro* and *in vivo* lung infection models using monoclonal antibodies

**DOI:** 10.1080/21505594.2018.1520545

**Published:** 2018-10-05

**Authors:** Philipp Janesch, Harald Rouha, Adriana Badarau, Lukas Stulik, Irina Mirkina, Marisa Caccamo, Katharina Havlicek, Barbara Maierhofer, Susanne Weber, Karin Groß, Jacqueline Steinhäuser, Manuel Zerbs, Cecilia Varga, Ivana Dolezilkova, Sabine Maier, Gerhild Zauner, Nels Nielson, Christine A. Power, Eszter Nagy

**Affiliations:** a Arsanis Biosciences, Vienna, Austria; b Adimab LLC, Lebanon, NH, USA

**Keywords:** *Streptococcus pneumoniae*, neuraminidases NanA NanB NanC, human and mouse lung infection models, host-pathogen interactions, monoclonal antibodies

## Abstract

*Streptococcus pneumoniae* isolates express up to three neuraminidases (sialidases), NanA, NanB and NanC, all of which cleave the terminal sialic acid of glycan-structures that decorate host cell surfaces. Most research has focused on the role of NanA with limited investigations evaluating the roles of all three neuraminidases in host-pathogen interactions. We generated two highly potent monoclonal antibodies (mAbs), one that blocks the enzymatic activity of NanA and one cross-neutralizing NanB and NanC. Total neuraminidase activity of clinical *S. pneumoniae* isolates could be inhibited by this mAb combination in enzymatic assays. To detect desialylation of cell surfaces by pneumococcal neuraminidases, primary human tracheal/bronchial mucocilial epithelial tissues were infected with *S. pneumoniae* and stained with peanut lectin. Simultaneous targeting of the neuraminidases was required to prevent desialylation, suggesting that inhibition of NanA alone is not sufficient to preserve terminal lung glycans. Importantly, we also found that all three neuraminidases increased the interaction of *S. pneumoniae* with human airway epithelial cells. Lectin-staining of lung tissues of mice pre-treated with mAbs before intranasal challenge with *S. pneumoniae* confirmed that both anti-NanA and anti-NanBC mAbs were required to effectively block desialylation of the respiratory epithelium *in vivo*. Despite this, no effect on survival, reduction in pulmonary bacterial load, or significant changes in cytokine responses were observed. This suggests that neuraminidases have no pivotal role in this murine pneumonia model that is induced by high bacterial challenge inocula and does not progress from colonization as it happens in the human host.

## Introduction

*Streptococcus pneumoniae* (the pneumococcus) is one of the most common human pathogens. Transmitted by aerosols, *S. pneumoniae* colonizes the nasopharynx, the natural reservoir of this Gram-positive bacterium. From this site, bacteria can ascend to the sinuses (sinusitis) or the middle ear (otitis media). Pneumococcal spread to the lungs can cause pneumonia, followed by bacterial invasion into the bloodstream (bacteraemia) and ultimately penetration of the blood-brain barrier (meningitis), with disease severity increasing in this order [1]. The majority of deaths and the highest morbidities are generally observed among the elderly (> 65 years) and in children under the age of 5 years, especially in developing countries [2].

Based on the capsular polysaccharide composition, more than 90 pneumococcal serotypes have been identified. Vaccines have been developed that elicit immune responses against the most common capsular serotypes [1]. Since the introduction of these vaccines, a significant reduction in the rates of childhood pneumococcal disease has been observed. However, variable impacts of vaccination on invasive pneumococcal disease rates, such as bacteraemia, septicaemia or meningitis often combined with pneumonia, have been reported in the elderly [3,4]. Rates of non-bacteraemic pneumococcal pneumonia in this age group have remained largely unaffected [5–7]. Present challenges in combating *S. pneumoniae* infections include serotype replacement (i.e. strains covered by vaccines are being substituted by non-vaccine serotypes), different world-wide serotype distribution patterns, recently described emergence of pathogenic non-encapsulated strains and increasing antibiotic resistance [8–10]. Additionally, despite proper antibiotic treatment, mortality rates are high, especially in the elderly. This is thought to be caused, at least partly, by damaging inflammatory host responses induced by the pneumococcus. Therefore, anti-virulence approaches, such as those based on monoclonal antibodies (mAbs) targeting pneumococcal virulence factors are being considered to improve therapeutic efficacy [11–16].

Pneumococcal fitness relies heavily on the glycolytic metabolism [17]. However, the availability of free hexoses (such as glucose) in the healthy human respiratory tract is limited [18]. Therefore, different strategies have evolved in pneumococci to gain access to nutrient sources. One such mechanism is the utilization of sugars that decorate host cell surfaces, such as sialic acids. Neuraminidases (sialidases) are key enzymes in sugar acquisition. Three pneumococcal neuraminidases, NanA, NanB and NanC have been described [19–21], with reported gene frequencies of 100, 96 and 36–51% among clinical isolates, respectively [22,23]. Neuraminidases share critical sequence features, such as the N-terminal signal peptide, a carbohydrate binding module (CBM40) that recognizes sialic acid (lectin-binding domain), and a six-bladed β-propeller catalytic domain (sialidase domain) with an inserted domain of unknown function [24,25]. NanA (115 kDa) additionally has a C-terminal LPETG membrane anchor motif, which is absent in NanB (78 kDa) and NanC (82 kDa). NanB shares approximately half of its amino-acid residues with NanC, but only one quarter with NanA. Differences in hydrophobicity around the active sites exist, whereas residues required for catalysis, such as a tri-arginine cluster that interacts with sialic acid, are conserved. All three neuraminidases recognize α2,3-linked sialic acids, but only NanA shows considerable activity towards α2,6-, and α2,8-linkages [21,24–27].

NanA and NanB were shown to cleave terminal sialic acid from host glycoconjugates (desialylation), which enables other exoglycosidases to subsequently remove underlying sugars to provide key nutrients for the bacterium [28,29]. NanA is required for pneumococcal growth on mucins (sialylated glycoproteins abundantly found in the respiratory tract) [30] and its expression is upregulated in the presence of lung cells [31]. By deglycosylating human serum components, NanA can interfere with C3 deposition on the pneumococcal surface and reduce opsonophagocytosis by human neutrophils [32]. Moreover, sequential deglycosylation can expose otherwise hidden receptors, such as asialo-GM1, which was shown to act as a receptor for pneumococcal binding to the host epithelium [33–35]. NanA, NanB and the presence of sialic acid can contribute to biofilm formation *in vitro* [36–38].

Both enzymes were demonstrated to be involved in colonization and infection of the upper and lower murine respiratory tract [39,40], although no defect in murine colonization was reported by others using a pneumococcal strain deficient in *nanA, bgaA* and *strH* [28]. Additional roles of NanA during persistance in the chinchilla middle ear infection model were shown [41–43]. NanA is required for adherence and invasion of brain endothelial cells, indicating its involvement in the development of meningitis [44]. The role of NanA during sepsis is more controversial, with reports ranging from greatly attenuated virulence of *S. pneumoniae nanA* gene deletion strains to no effect [40,45].

Removal of host surface glycan-structures by NanA can induce pro-inflammatory responses, leading to recruitment of immune cells, which can be detrimental if uncontrolled [46].

The contribution of NanC to pneumococcal disease is not well understood. Although the presence of *nanC* has initially been suggested to be linked to *S. pneumoniae* isolates from cerebrospinal fluid [22], this finding could not be confirmed in a more recent study [47]. In addition, pneumococcal isolates harbouring *nanC* have been implicated to be involved in necrotizing pneumonia and subsequent development of haemolytic uremic syndrome (HUS) [48], a finding that could not be confirmed by another group [49].

NanA, NanB and NanC can cleave serum sialoglycoproteins, including fetuin-A, low levels of which were associated with invasive pneumococcal disease and HUS [50]. Notably, immunization with all three recombinant neuraminidases was shown to modestly increase survival of mice challenged intravenously with different *S. pneumoniae* strains [51].

Given the multiple functions of neuraminidases in the pneumococcal life cycle and their complex interactions with the host, we hypothesized that inhibiting the activity of NanA, NanB and NanC by monoclonal antibodies could provide novel insights into the biological roles of these virulence factors and complement existing knowledge that has hitherto been generated mainly with recombinant neuraminidases or gene deletion mutant strains.

## Materials and methods

### Cloning, expression and purification of recombinant neuraminidases

Published *S. pneumoniae* D39 NanA lectin domain (LD) and sialidase domain (SD) protein sequences (GenBank: ABJ55437.1, aa 16–279 and 280–756, respectively) were blasted against the NCBI database. A total of 310 unique protein sequences were analysed and for each domain, two clade variants were identified. Inter-clade sequence conservation was ~ 83 and 82%, while intra-clade sequence conservation was > 94 and 96% for the LD and SD, respectively. Three NanA recombinant proteins containing LD and SD from different clades (NanA-LD-SD, NanA-LDvar-SD, NanA-LD-SDvar) but missing the C-terminal hydrophobic region (downstream of the LPXTG anchor motif), and also NanB and NanC proteins (Table 1), were cloned in mature form (signal peptide predicted with SignalP 4.0 [52]), without tags, in pET24a, and overexpressed in *E. coli* in soluble form. Chromatographic protein purification was performed using anion exchange (HiTrap Q Sepharose FF, GE Healthcare) with a salt gradient, followed by anion exchange (HiTrap Q Sepharose HP, GE Healthcare) with a pH gradient and size exclusion (Superdex 200, 16/60, GE Healthcare). Purity and monomeric content for all proteins, both > 95%, were determined by SDS-PAGE, size exclusion chromatography and circular dichroism for the secondary structure. Enzymatic activities (k_cat_/K_m_) were determined using a fluorometric assay as described previously [53]. NanA baits for antibody library selections were generated by biotinylation using an amine reactive reagent (Sulfo-NHS-LC biotin, Pierce) according to manufacturer’s instructions.

**Table 1.** Recombinant proteins used in this study. Neuraminidase protein characteristics are outlined in the table below.

protein name	strain	GenBank	domains removed	characteristics
NanA-LD-SD	D39	ABJ55437.1	signal peptide, C-terminus	“wild-type” sequence
NanA-LDvar-SD	70,585	ACO17645.1	signal peptide, C-terminus	most amino acid variations in lectin domain (LD)
NanA-LD-SDvar	INV200	CBW35147.1	signal peptide, C-terminus	most amino acid variations in sialidase domain (SD)
NanB	D39	ABJ55283.1	signal peptide	
NanC	TIGR4	AAK75424.1	signal peptide	

### Pneumococcal strains, lysates and culture supernatants

Clinical *S. pneumoniae* isolates collected in eight hospitals in Hungary between 2000 and 2008 from patients (aged between 8 months to 74 years) with invasive infections or pneumonia [54] were kindly provided by Orsolya Dobay (Semmelweis University, Budapest, Hungary). The strains D39, A66.1 and EF3030 were kindly provided by David Briles (University of Alabama at Birmingham, AL, USA); TIGR4 was obtained from ATCC (BAA-334™). Strain characteristics are outlined in Table S1.

Bacterial lysates were generated from cultures grown in THY medium (Todd Hewitt Broth + 0.5% yeast extract; Sigma) to OD_620nm_ of 0.3 (37°C, 5% CO_2_), pelleted, washed with PBS and homogenized using 2 mL tubes containing glass beads (Precellys® glass kit 0.1 mm) in a Precellys 24 tissue homogenizer. Total protein concentration of lysates was measured with a BCA kit (Pierce), according to the manufacturer’s instructions.

Culture supernatants (CS) were generated from strains grown in THY medium to early stationary phase (37°C, 5% CO_2_) as previously described [55]. Cultures were centrifuged (5.000 x g, 10 min, 4°C), CS were harvested, sterile filtered (0.1 μm Millex® PVDF syringe-driven filter units, Millipore) and concentrated 15-fold (Vivaspin®20 centrifugal concentrators, MWCO = 10 kDa, Sartorius).

### Generation of S. pneumoniae gene deletion mutant strains

Isogenic *S. pneumoniae* gene deletion mutant strains (Δ*ply*, Δ*nanA*, Δ*nanB*, Δ*nanA*Δ*nanB*) were generated by allelic replacement in the D39 background as previously described [56], selecting for Ery^R^ or Kan^R^ clones on Columbia agar supplemented with 5% sheep blood (COS, Biomerieux) and antibiotics (0.3 μg/mL erythromycin, or 400 μg/mL kanamycin). Primers used for mutagenesis are listed in Table S2. Competence in D39 was induced by CSP-1 (Eurogentec) in the presence of 1% CaCl_2_. Gene-deletions were verified by PCR, lack of functional activity, or immunoblotting (neuraminidases and pneumolysin).

Growth rates of mutants in THY medium (37°C, 5% CO_2_) did not differ from the D39 parental strain. Deletion of *nanA* did not result in altered NanB expression tested with strains grown to early stationary phase in THY medium (37°C, 5% CO_2_). Similarly, no effect of *nanB* deletion on NanA expression could be detected (based on immunoblot analysis).

### DNA isolation, neuraminidase gene screening and genotyping of clinical *S. pneumoniae isolates*

Pneumococcal genomic DNA was isolated using the DNeasy Blood and Tissue Kit 250 (Qiagen) according to the manufacturer’s instructions. Detection of *nanA, nanB* and *nanC* gene fragments (~ 500 bp) was performed by PCR as described previously [22]. Neuraminidase genes (~ 3000 bp) were amplified using primers 31R and 31S [57] (*nanA*), B_seq1 and B_seq8 (*nanB*), or C_seq1 and C_seq8 (*nanC*); genes encoding pneumolysin (Ply) and PspA clade defining regions as described elsewhere [58–60]. Amplicons were sequenced using primers listed in Table S3. Sequences were assembled and aligned using the CLC Main Workbench 6 software (Qiagen).

### Generation of anti-NanA mAbs

NanA-specific mAbs were generated by *in vitro* selection using full length human IgG1 libraries expressed by yeast as previously published [61–64] and biotinylated NanA-variants as baits. Antibody clones were characterized as described below and selected for affinity-maturation (light chain diversification and subsequent heavy chain mutagenesis).

VH and Vk sequences of selected mAbs were cloned into pTT5 mammalian expression vectors (Biotechnology Research Institute, National Research Council of Canada, Quebec) in-frame with human IgG1/human kappa light chain constant regions. MAbs were expressed in CHO-3E7 cells (Biotechnology Research Institute, National Research Council of Canada, Quebec) by co-transfecting with pTT5 plasmids encoding heavy and light chains using PEI MAX (Polysciences) for DNA delivery. MAbs were purified from supernatants 8 days post transfection using HiTrap MAbSelect or MabSelectSuRe (for the mAbs of human VH3 germline) pre-packed columns (GE Healthcare).

### Generation of anti-NanBC cross-reactive mAbs

Mice were subcutaneously immunized three times with recombinant NanB (10 µg/mouse/immunization, formulated with Alum or Freund’s adjuvant) and hyperimmune sera were tested for cross-reactivity against NanC. The mouse with the highest cross-neutralizing titer was intravenously boosted with NanC (5 µg) for subsequent hybridoma generation according to standard protocols.

Selected hybridoma clones expressing mAbs that neutralized both enzymes were subjected to sequencing of antibody heavy and light chain genes. Briefly, mRNAs isolated from these clones were reverse-transcribed with AccuScript^TM^ High Fidelity Reverse Transcriptase and oligo (dT) primers (Agilent). MAb VH and Vk sequences were amplified from the respective cDNAs using heavy and light chain primer mixes (GE Healthcare) and cloned into a pJET1.2 vector (Fermentas) for sequencing with vector-specific primers. Sequences were analyzed against the mouse VH and Vk germline repertoire with IMGT V-QUEST [65] to determine germline type, presence of somatic mutations, position and length of the CDRs and framework regions.

To generate chimeric mAbs, the identified mouse VH and Vk were fused with human IgG1/human kappa light chain constant regions in a pTT5 vector. MAbs were produced in CHO-3E7 cells as described above.

### Binding affinity determination of mAbs

For the affinity determination of α-NanA mAbs, binding of the Fab fragments to NanA and its sequence variants was measured by biolayer interferometry using a ForteBio Octet Red Instrument (Pall Life Sciences). The biotinylated antigen was immobilized on the streptavidin sensor and the association and dissociation of the Fab fragment (100 nM), in solution (PBS, pH 7.2 + 1% BSA), were monitored for 3 min each. The monovalent affinities expressed as dissociation constants (K_D_ values) were calculated based on the measured kinetic parameters (k_on_ and k_off_) by simultaneously fitting the association and dissociation phases to a 1:1 binding model using Octet Data Analysis Software version 7. In an alternative setup the mAb was immobilized on an anti-human capture (AHC) sensor and the association and dissociation of the antigen (200 nM for NanA-SD or NanA-LD), in solution (PBS, pH 7.5 + 0.1% BSA), at 30°C, were monitored for 5 and 3 min, respectively. Affinities of α-NanBC mAbs for NanB and NanC were also measured by biolayer interferometry: the chimeric mAb was immobilized on the AHC sensor and the association and dissociation of the antigen (100 nM), in solution (PBS, pH 7.5 + 0.1% BSA), at 30°C, were monitored for 10 min each. Monovalent affinities and kinetic parameters were determined as for NanA.

### *In vitro* neuraminidase enzymatic assay

Neuraminidase activity was measured in a fluorometric assay using the sialic acid analogue 2′-(4-Methylumbelliferyl)-α-D-N-acetylneuraminic acid sodium salt hydrate (NEU5AC-A-4MU, Sigma) as substrate [53] in HBSS + 0.5% BSA buffer. The fluorescent (λ_ex_ = 365 nm, λ_em_ = 450 nm) signal emitted upon substrate cleavage by pneumococcal neuraminidases was detected using a BioTek-Synergy HT plate reader.

Assay conditions were adapted to measure neuraminidase activity of recombinant neuraminidases, pneumococcal lysates or CS in the presence of α-neuraminidase mAbs as specified in the corresponding figure legends. Data were collected at 5–30 min, 4 h, or 10 h after addition of the substrate (200–400 µM), respectively.

### Human cell surface desialylation assay

Raji cells (ATCC® CCL86™) used at 2.5 to 5 × 10^5^ cells/well were incubated with recombinant NanA (1 nM) at 4°C for 30 min or infected with *S. pneumoniae* D39Δ*ply* at a multiplicity of infection (MOI) 10 for 3 h at 37°C in RPMI (Gibco, Thermo Fisher Scientific) + 10% FCS (Sigma). Inhibition of cell surface desialylation by α-NanA mAbs (4 – 1000 nM) was measured by flow cytometry (BD Accuri) using anti-asialo-GM1 polyclonal rabbit IgG (eBioscience) and Alexa Fluor® 488 F(ab’)2 fragment of goat anti-rabbit IgG H + L (Molecular Probes) as secondary reagent, or an *A. hypogaea* (peanut) lectin FITC conjugate (Sigma).

### In vitro infection assay using the A549 lung epithelial cell line

*S. pneumoniae* strains were grown in THY medium to OD_620nm_ of 0.3, pelleted, washed with PBS and re-suspended in antibiotic-free F12K medium (Invitrogen) + 10% FCS (Sigma). A human pulmonary epithelial cell line (A549; ATCC® CCL-185™; adenocarcinomic alveolar basal epithelial cells), grown to confluent monolayers in the same medium, was infected at MOI 10 for 48 h (for D39 and mutants), 3 or 24 h (for EF3030 and Sp#5) at 37°C, 5% CO_2_.

At the end of infection supernatants were harvested (to collect unbound bacteria), A549 cells were washed twice with PBS, detached and lysed using Trypsin/EDTA (Invitrogen) and 2% saponin (Sigma), respectively. Serial dilutions of bacteria in the supernatant and those recovered from A549 cells were plated onto Columbia agar plates supplemented with 5% sheep blood (COS, Biomerieux) and incubated overnight (37°C, 5% CO_2_) for CFU enumeration.

### Human 3D mucociliary lung tissue model

Multi-layered, mucus-producing normal human tracheal/bronchial lung epithelial cells (EpiAirway™, MatTek, 24- or 96-well) were processed according to the supplier’s protocol, cultured and infected at the air-liquid interface.

Pneumococcal strains were prepared as above and re-suspended in antibiotic-free medium (MatTek). To study desialylation, tissues were infected with D39Δ*ply* (MOI 100) or treated with recombinant neuraminidases. Alternatively, tissues were infected with *S. pneumoniae* EF3030 or Sp#5 at MOI 5 in the presence of α-neuraminidase and α-Ply mAbs. Tissues were incubated for 24 h (37°C, 5% CO_2_) and subsequently fixed (3 h at room temperature) with 10% neutral buffered formalin solution (Sigma) for fluorescence microscopy.

Additionally, the bacterial interaction with the human lung tissue was evaluated from experiments with the EF3030 or Sp#5 strains. Supernatants of infected samples were collected at the end of the incubation time (to harvest unbound bacteria), tissues were washed twice with PBS, removed using sterile tissue punches (3.5 mm, Integra, Miltex), homogenized with a Precellys 24 homogenizer (Precellys bulk beads 1.4 mm Zirconium oxide) and treated with 2% saponin (Sigma) for subsequent CFU enumeration as described above.

### Murine *S. pneumoniae* pneumonia models

Animal experiments were performed according to Austrian Law (BGBl. I Nr. 114/2012, approved by MA58, Vienna, Austria) and IACUC approval, with groups of 5 female, 6–8 week- old C57BL/6JRj mice (Janvier, France). MAbs (200–300 µg/mAb/mouse, formulated in PBS) were given by intraperitoneal injection, 48 h prior to intranasal bacterial challenge. Control groups received an isotype-matched (human IgG1) control mAb. Mice were anesthetized with ketamine/xylazine followed by drop-wise intranasal application of 40 µL of the bacterial challenge inoculum (2-3x10^7^ CFU/mouse). Survival was monitored for up to 10 days post challenge or animals were sacrificed after 24 or 48 h for the determination of bacterial organ loads (enumeration of bacterial CFUs in serial fold-dilutions of the respective organ after plating on COS agar plates and incubation at 37°C, 5% CO_2_, > 16 h) or for staining of lung tissues. Statistical analyses were performed with the log-rank (Mantel-Cox) test using GraphPad Prism 6 software.

### Tissue staining to detect desialylation

Formalin-fixed lung tissues and mouse lungs were embedded in paraffin to generate histological sections of 3 μm, or 1 μm thickness respectively. Samples were stained with *A. hypogaea* (peanut) lectin-FITC conjugate (10 μg/mL) to detect desialylation (Sigma) and Hoechst 33,258 (Sigma) to stain cell nuclei. Samples were visualized with an Olympus VS120 S6-E microscope (VS-XM10tR camera, UPLSAPO40x objective NA = 0.95) and analysed with OlyVIA 2.9 software. Background was removed without obscuring biological information.

A semi-quantitative scoring system was employed to assess the extent of desialylation by microscopic analysis of lungs of mice infected intranasally with *S. pneumoniae* in presence of mAbs as follows: score 2 = desialylation, score 1 = reduced desialylation, or score 0 = no desialylation. Conducting zones (comprising main bronchi and terminal bronchioles) and respiratory zones (comprising respiratory bronchioles, alveolar areas and interstitial cells) were scored independently. Cumulative scores from ten mice per treatment group (maximum score = 20, minimum score = 0) were generated.

### Determination of cytokine and chemokine levels in lung tissue

Lungs of mice infected with *S. pneumoniae* EF3030 or Sp#5 in presence of α-neuraminidase or control mAbs were excised, washed in PBS and weighed before homogenization (Precellys 24 homogenizer, Precellys bulk beads 1.4 mm Zirconium oxide). Proteinase inhibitor cocktail (Sigma) was added to the homogenized samples, which were centrifuged (16.000 x g, 15 min, 4°C) to harvest supernatants (stored at −80°C until use).

Cytokine and chemokine levels in supernatants were determined using the Mouse Inflammation Panel and Mouse Proinflammatory Chemokine Panel (LEGENDplex™ multiplex cytokine bead arrays; BioLegend) according to the manufacturer’s instructions. Samples were measured on a CytoFLEX flow cytometer (Beckman Coulter) and results were analysed using LEGENDPlex 7.0 software (BioLegend). Analyte levels were normalized to the weights of lung tissues. The analytes and detection limits were as follows: RANTES/CCL5 (0.76 pg/mL), MIP-3α/CCL20 (1.20 pg/mL), Eotaxin/CCL11 (1.39 pg/mL), TARC/CCL17 (1.28 pg/mL), KC/CXCL1 (1.47 pg/mL), MCP-1/CCL2 (1.13 pg/mL), MIG/CXCL9 (1.34 pg/mL), IP-10/CXCL10 (0.63 pg/mL), MIP-1α/CCL3 (1.58 pg/mL), MIP-1β/CCL4 (1.56 pg/mL), BLC/CXCL13 (1.50 pg/mL), LIX/CXCL5 (9.77 pg/mL), MDC/CCL22 (1.13 pg/mL), IL-23 (2.50 pg/mL), IL-1α (0.53 pg/mL), IFN-γ (0.89 pg/mL), TNF-α (1.33 pg/mL), IL-12p70 (0.70 pg/mL), IL-1β (1.84 pg/mL), IL-10 (1.16 pg/mL), IL-6 (0.53 pg/mL), IL-27 (34.22 pg/mL), IL-17A (0.67 pg/mL), IFN-β (8.64 pg/mL), GM-CSF (1.26 pg/mL).

## Results

### Generation of α-NanA mAbs to inhibit the activity of different NanA variants

**Figure 1.**
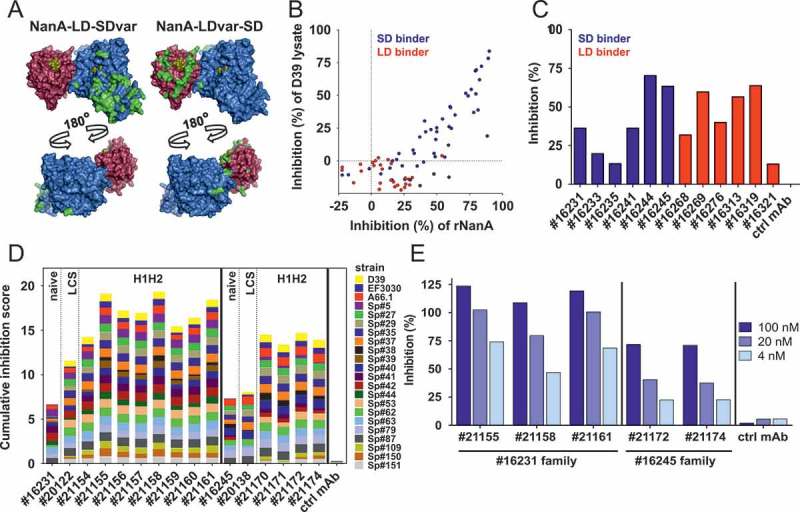
Generation of α-NanA mAbs. (A) Sialidase (blue, PDB 3H72 [26]) and lectin (red, PDB 4C1X [66]) domains of NanA aligned (using PyMOL software v2.0.5; Schrödinger) on the NanB structure (PDB 2VW1 [25]) as a scaffold. Residues differing from NanA-LD-SD are highlighted in green, and sugars occupying the sugar binding sites are shown in yellow. (B) Inhibition of neuraminidase activity of recombinant NanA (2.5 nM) and NanA from D39 lysates (used at a total protein concentration of 20 μg/mL) by naïve yeast library derived α-NanA mAbs (1 or 0.33 µM, respectively) in a fluorometric assay. (C) Inhibition of 1 nM rNanA-mediated Raji cell surface desialylation by naïve α-NanA mAbs (1 µM). (D) Cumulative inhibition of NanA sialidase activity in pneumococcal lysates (20–40 μg/mL) by selected naïve, and LCS (light chain shuffled) or mutated HCDR1 + HCDR2 (H1H2) SD-binding mAbs (0.33 µM) in a fluorometric assay. Inhibition scores for each lysate from 0 (no inhibition) to 1 (100% inhibition) were calculated relative to a control human IgG1. Cumulative inhibition scores were generated by addition of individual values (minimum score = 0, maximum score = 22). (E) Inhibition of Raji cell surface desialylation during infection with *S. pneumoniae* (D39Δ*ply*) by H1H2 α-NanA mAbs at the indicated concentrations. One representative experiment from the original screening data is shown in panels B, C and E. Two independent experiments are summarized in panel D.

NanA contains two main functional domains, the lectin-binding domain (LD) and the sialidase domain (SD). Amino acid sequence alignment of publicly available NanA sequences (n = 310) allowed clustering into three main groups: NanA-LD-SD (represented by strain D39), NanA-LDvar-SD (variant LD domain, but SD conserved with prototype strain D39, example strain 70,585) and NanA-LD-SDvar (D39 like LD domain with variant lectin domain, example strain INV200). These three variants represented 74, 5 and 13% of published NanA sequences, respectively, and cover > 90% of NanA diversity. This grouping therefore also provided the basis for antigen expression and all three bait types were used for antibody selection. The highest NanA amino acid sequence diversity was observed in the regions around the sugar binding site of the LD and the insertion sequence of the SD (Figure 1(A)).

To cover a broad spectrum of pneumococcal diversity and to exclude strain-specific effects during mAb generation, we chose 20 highly diverse clinical *S. pneumoniae* isolates and four reference strains (D39, A66.1, EF3030 and TIGR4) based on serotype, PspA clade and *ply* allele (strain characteristics are shown in Table S1). Gene frequencies for *nanA, nanB* and *nanC* were 100, 96 and 38%, respectively among these strains.

The overall amino acid identities of NanA in this set of strains (without the signal peptide) was determined to be ≥ 83% (excluding TIGR4, which encodes a truncated NanA). Three NanA clusters could be identified in line with the representative variants from published sequences (Figure S1). The overall mean distance (Poisson model, Gamma distribution) between the three NanA clusters was 0.066 as opposed to the within group distances of 0.012 (cluster 1), 0.009 (cluster 2), or 0.005 (cluster 3). No variations of amino acid residues previously determined as essential for enzymatic activity were identified in these strains [67].

Using yeast expression libraries of human IgG1 sequences, we selected approximately 80 mAbs binding to either the SD or LD based on biolayer interferometry measurements and immunoblotting with the respective recombinant domains. Although only the SD-binding mAb fraction showed inhibitory activity of NanA with a synthetic substrate in the fluorescence assays (Figure 1B), both SD- and LD-binding mAbs were able to block desialylation of eukaryotic cells (Figure 1C), indicating activity against the natural substrate for both mAb types. The most potent “näive” mAbs were subjected to affinity maturation with light chain shuffled and HCDR1 and HCDR2 (H1H2) mutated sub-libraries. Next, we selected mAbs with high inhibitory potential and broad reactivity against NanA sequence variants, tested with the panel of pre-characterized *S. pneumoniae* isolates (Figure 1D)). Based on the cumulative inhibition of NanA activity of these strains, as well as highest neutralizing potency against the major NanA variant (NanA-LD-SD) upon infection of Raji cells with *S. pneumoniae* D39Δ*ply* (to avoid cell lysis) (Figure 1E)), mAb #21155, a SD-binder, was selected for further studies. Binding affinities of this mAb to NanA-LD-SD, NanA-LDvar-SD and NanA-LD-SDvar were in the low nanomolar range (Table S4). No cross-reactivity of this mAb to NanB or NanC could be detected.

### Generation of cross-reactive α-NanBC mAbs

**Figure 2.**
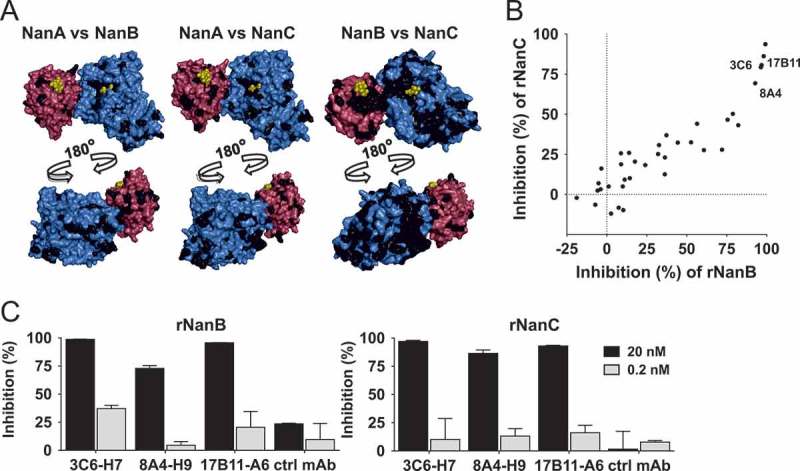
Discovery of cross-reactive α-NanBC mAbs. (A) Conservation among NanA, NanB and NanC. NanA structure as depicted in Figure 1, with residues identical in NanB (left panel) and NanC (4YC4 [27], middle panel) marked in black, and NanB (PDB 2VW1 [25], right panel, colors as for NanA) with residues identical in NanC marked in black. (B) Mouse hybridoma mixed clone supernatants tested for neutralization of recombinant NanB and NanC (2 nM each) in a fluorometric screening assay. One representative experiment is shown. (C) Three human-mouse chimeric mAbs expressed in CHO cells, tested at the indicated concentrations for inhibition of NanB and NanC in two independent experiments shown as mean with standard deviation.

Sequencing of *nanB* and *nanC* from the 24 pre-selected *S. pneumoniae* strains (Table S1) revealed ≥97% conservation at the amino acid level for both NanB and NanC. Protein identities between NanB and NanC were approximately 46%, and to NanA (without C-terminal anchor) approximately 20%. The majority of the few conserved residues between NanA and the other two neuraminidases were located around the sugar binding site of the SD. In contrast, NanB and NanC showed high sequence conservation around the sugar binding sites of both the SD and the LD, as well as the region opposite to the SD active center (Figure 2A).

We aimed at generating cross-reactive α-NanBC mAbs using a murine hybridoma approach employing alternating immunization of mice with both antigens. Mixed clone hybridoma supernatants (n = 1920) were tested for binding to NanB and NanC (by ELISA), followed by screening for neutralization of enzymatic activities using recombinant proteins (Figure 2B)). Several clones showed cross-reactivity and cross-neutralization in the single clone stage and the five most potent hybridoma clones were subjected to DNA sequencing. Three of them had unique VH and Vk sequences and were expressed as mouse-human chimeric antibodies. Based on its highest *in vitro* potency, mAb 3C6-H7 was selected for further studies (Figure 2C)). Binding affinities of this mAb to NanB and NanC were in the picomolar range (Table S4). No cross-reactivity of 3C6-H7 to NanA could be detected.

## Contribution of the three neuraminidases to the total neuraminidase activity varies substantially among different *S. pneumoniae isolates*

**Figure 3.**
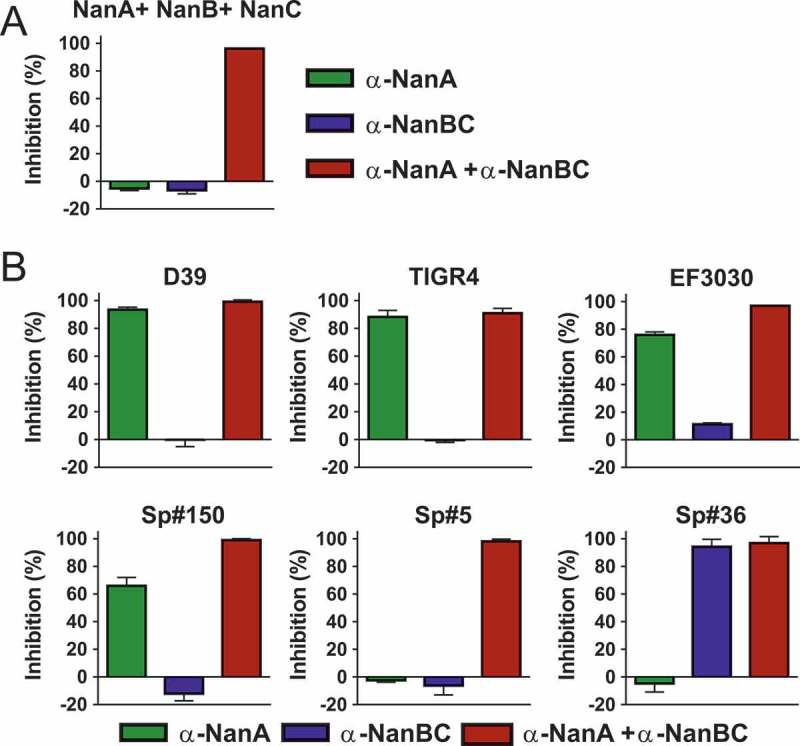
Contribution of individual Nans to the total neuraminidase enzymatic activity differs among *S. pneumoniae* strains. Neuraminidase activity of recombinant neuraminidases (200 pM each) (A) or pneumococcal stationary phase CSs (B) in the presence of α-neuraminidase mAbs (1 µM each). Two clones per strain were used for CS generation. Percent inhibition was calculated relative to a negative control mAb. Results from two independent experiments are shown as mean with standard deviation.

The activity and contribution of NanA vs. NanB and/or NanC to the total pneumococcal neuraminidase activity was addressed in the enzymatic fluorescent assay using pneumococcal early stationary phase culture supernatants (CS) of six *S. pneumoniae* strains in the presence of the α-NanA and α-NanBC mAbs (Figure 3). Since NanB and NanC are secreted proteins, these experiments were performed with CS, where NanA is also present (despite being mainly surface anchored, except for the TIGR4 strain). This *in vitro* model was validated by showing complete inhibition of the enzymatic activity of an equimolar mixture of the three recombinant neuraminidases (200 pM each) by α-NanA and α-NanBC mAbs (1 µM each) (Figure 3A)). Importantly, the neuraminidase activities of the CSs at the tested dilution were lower in this assay compared to those of the recombinant proteins inhibited with the same amount of mAbs.

Based on the inhibition data collected, the tested isolates could be grouped into three categories (Figure 3B)): In the first category neuraminidase activity was solely mediated by NanA since full inhibition was obtained with the α-NanA mAb (D39, TIGR4). In a second group of strains, NanB and/or NanC activity was detectable on top of NanA (EF3030, Sp#150 and Sp#5). Neither antibody alone could fully prevent substrate cleavage, but complete inhibition was observed with the mAb combination. The different inhibition levels in presence of the α-NanA mAbs in this category suggest different levels of NanB/C activity. In a third category (Sp#36), we observed NanB/C dependent cleavage and lack of NanA activity (full inhibition with the α-NanBC mAb). Sequence analysis of this particular strain revealed a frameshift mutation resulting in a truncated NanA protein (only 74 amino acids long). To our knowledge this is the first reported naturally occurring *S. pneumoniae* strain that does not produce functional NanA.

These data suggest that the total neuraminidase activity in different *S. pneumoniae* isolates is highly variable and can involve one or up to all three neuraminidases. Efficient and complete inhibition of enzymatic sialidase activity in clinical *S. pneumoniae* isolates therefore necessitates simultaneous targeting of all three neuraminidases.

### Pneumococcal neuraminidases desialylate human lung tissue in vitro

**Figure 4.**
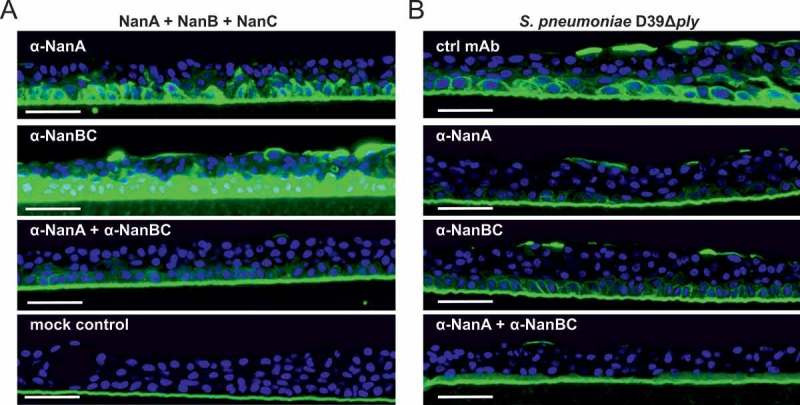
Multiple neuraminidases contribute to desialylation of primary human lung tissue by *S. pneumoniae*. Primary human lung tissue treated with an equimolar mixture of all three recombinant neuraminidases (5 nM each) ± α-neuraminidase mAbs (2 µM each) (A) or infected with *S. pneumoniae* D39Δ*ply* +/- α-neuraminidase mAbs (2 µM each) or control mAb (4 µM) (B) for 24 h. Desialylation was visualized by Peanut Lectin-FITC staining (green). Nuclei were stained with Hoechst dye (blue). Representative images of one experiment with biological duplicates are shown. Scale bar = 50 μm.

To mimic a relevant *in vivo*-like situation, we employed an *in vitro* lung infection model, in which a human, three-dimensional multi-layered and mucociliated airway epithelial primary tissue was infected with *S. pneumoniae*. The activity of the *in situ* produced neuraminidases was tested by detecting cellular desialylation, and their contributions were dissected by neutralization with the α-NanA and/or α-NanBC mAbs. We used a staining method that employed fluorescently labelled peanut lectin which specifically recognizes the sugar moiety Galβ3GalNAc that only becomes unmasked after neuraminidase-mediated removal of terminal sialic acids. First, we confirmed that each individual recombinant neuraminidase was able to desialylate human lung airway epithelial cells in this tissue model (data not shown). Desialylation by an equimolar mixture of all three recombinant neuraminidases was greatly reduced in the presence of the mAb-combination but only partially by the individual mAbs (Figure 4a). In agreement with this, prevention of desialylation during infection with *S. pneumoniae* D39Δ*ply* required both α-neuraminidase mAbs (Figure 4B)). Similar results were obtained with two other *S. pneumoniae* strains, EF3030 or Sp#5 (data not shown).

These data suggest that pneumococcal neuraminidases are active towards the human airway epithelium and that α-neuraminidase mAbs can preserve the terminal epithelial sugar composition during *S. pneumoniae* infection.

### Pneumococcal neuraminidases enhance *S. pneumoniae* interaction with human epithelial cells

We further evaluated the contribution of individual pneumococcal neuraminidases to the interaction of *S. pneumoniae* with lung cells in several *in vitro* infection assays.

In a setup experiment, monolayers of human lung alveolar cells (A549) were infected with *S. pneumoniae* D39 or D39∆*nanA*∆*nanB* for 48 hours. Viability of infected compared to uninfected A549 cells was reduced (to approx. 75%). No differences in viability of A549 cells infected with either *S. pneumoniae* D39 or D39∆*nanA*∆*nanB* could be detected (data not shown).

**Figure 5.**
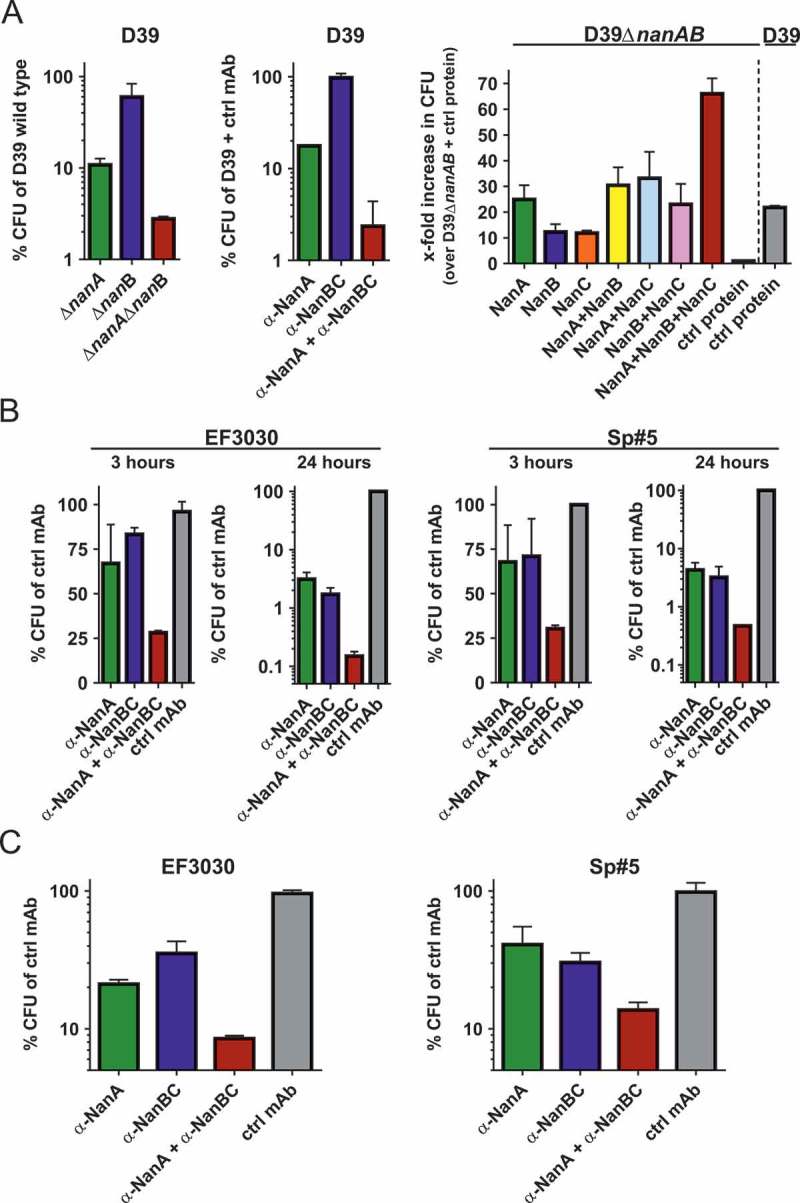
Neuraminidases increase pneumococcal interaction with human lung cells. (A) A549 cells infected with *S. pneumoniae* D39 and corresponding isogenic neuraminidase gene deletion mutants (left panel), or D39 wild-type in presence of α-neuraminidase mAbs (2 μM each) or 4 μM control IgG1 (middle panel). A549 cells treated with recombinant neuraminidases (1 nM each) or control protein (3 nM) and infected with D39Δ*nanA*Δ*nanB*, or D39 wild-type and control protein (right panel). B: A549 cells infected with EF3030 or Sp#5 strains in the presence of α-neuraminidase mAbs (2 µM each) or control IgG1 (4 µM). C: Primary human lung tissues infected with *S. pneumoniae* EF3030 or Sp#5 in the presence of α-Ply (2 µM) + α-neuraminidase mAbs (2 µM each) or α-Ply (2 µM) + control IgG1 (4 µM). CFUs recovered from infected cells were calculated relative to those obtained with comparator strains or treatments indicated on the y-axes. Results from two independent experiments with at least biological triplicates are depicted as mean with standard deviation.

Next, A549 cells were infected with *S. pneumoniae* D39 (*nanC* negative) or isogenic neuraminidase gene deletion mutants (Figure 5a, left panel) or wild-type D39 in presence of the α-NanA and α-NanBC mAbs (Figure 5A), middle panel). Removal of NanA (either by gene deletion or neutralization with the α-NanA mAb) reduced the number of bacteria in direct contact with A549 cells by approximately 10-fold, while removal of NanB had minimal effects. However, lack of both NanA and NanB reduced the recovered CFUs approximately 30 to 100-fold.

Reciprocally, D39Δ*nanA*Δ*nanB* was used to infect A549 cells in presence of recombinant neuraminidases (Figure 5A right panel). Addition of NanA increased the number of bacteria recovered from A549 cells by approximately 25-fold, whereas the addition of either NanB or NanC resulted in approximately 10-fold increased CFUs. Importantly, the presence of both NanB and NanC resulted in similar numbers of recovered bacteria as NanA alone. Addition of NanB or NanC on top of NanA did not enhance this effect, however, the presence of all three neuraminidases resulted in approximately 70-fold more bacteria associated with A549 cells, which could also be a consequence of the increased total neuraminidase amount. Samples treated with D39 wild-type and control protein showed similar CFUs as those infected with D39Δ*nanA*Δ*nanB* and treated with NanA, or NanA and a second neuraminidase.

Using two additional *S. pneumoniae* strains, EF3030 (*nanA/B*) and Sp#5 (*nanA/B/C*) in the same assay, more pronounced differences upon mAb-treatment were observed. Neutralization of NanB (EF3030), or neutralization of NanB and/or NanC (Sp#5) reduced bacterial numbers to the same level as seen with neutralization of NanA in a time-dependent fashion (Figure 5B). Simultaneous targeting of all neuraminidases further enhanced this effect, resulting in up to 3-log CFU reduction relative to the control group after 24 h.

In the multi-layered primary human airway epithelial tissue model, blocking of the neuraminidases by the two mAbs during EF3030 and Sp#5 infection resulted in highly similar effects on bacterial recovery (Figure 5C), although this was less pronounced (approximately 10 to 20-fold decrease).

In all abovementioned models, we also assessed the effect of neuraminidases on pneumococcal growth by CFU plating of bacteria recovered from supernatants. When A549 cells were infected for ≥24 h, the absence of neuraminidases resulted in up to 3 to 4-log reductions in bacterial growth and resembled changes observed with bacteria recovered from host cells. However, bacterial growth in the supernatants of A549 cells infected for 3 h, or primary human lung tissue infected for 24 h did not differ between experimental groups (data not shown).

These data suggest that all three neuraminidases enhance the interaction of pneumococci with human lung cells *in vitro* by increasing growth, or direct contact with host cells, or both.

### *Pneumococcal neuraminidases desialylate murine lungs during* S. pneumoniae *infection* in vivo

**Figure 6.**
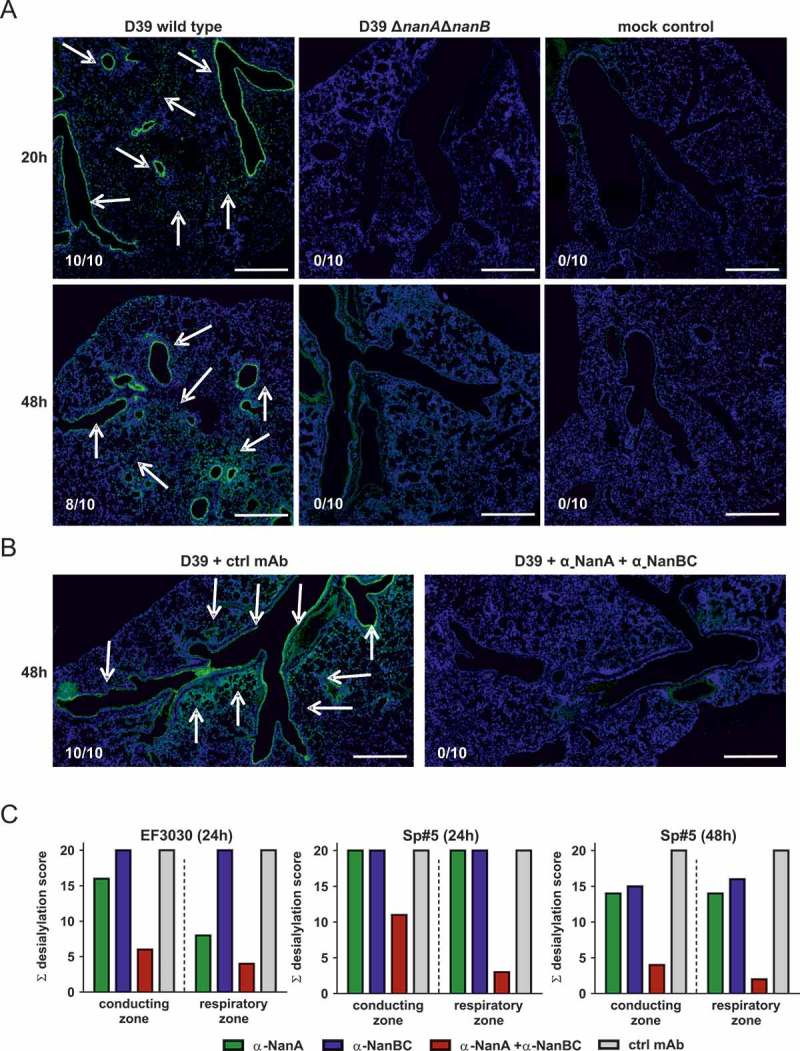
Neuraminidases of different *S. pneumoniae* isolates desialylate murine lungs *in vivo*. Mice infected intranasally with *S. pneumoniae* D39, or D39Δ*nanA*Δ*nanB* (A), or *S. pneumoniae* D39 + both α-neuraminidase mAbs (B), or *S. pneumoniae* EF3030 or Sp#5 in the presence of individual α-neuraminidase mAbs (c) for the indicated times. Arrows indicate desialylation as visualized by Lectin-FITC staining (green). Low levels of apparent signal for the Lectin-FITC staining were non-specific and also seen in uninfected controls. Nuclei were stained with Hoechst dye (blue). Representative lung images of each treatment group are shown. Numbers indicate the observed frequencies of desialylation. Scale bar = 500 μm. (C) Semi-quantitative scoring of conducting and respiratory zone desialylation by EF3030 and Sp#5 in presence of α-neuraminidase mAbs.

In order to investigate neuraminidase-mediated lung desialylation *in vivo*, mice were challenged intranasally with *S. pneumoniae* D39 wild-type (*nanA/B*) or D39Δ*nanA*Δ*nanB*. We observed strong desialylation of the conductive zones (comprising main bronchi and terminal bronchioles) and respiratory zones (respiratory bronchioles, alveolar areas, interstitial cells) upon challenge with the wild-type strain, but not with the neuraminidase-deficient strain (Figure 6a). Importantly, simultaneous neutralization of NanA and NanB by prophylactic administration of both α-neuraminidase mAbs abolished *S. pneumoniae* D39-mediated pulmonary desialylation (Figure 6B).

The contribution of NanA and NanB and/or NanC to desialylation was further assessed in experiments where mice were treated with α-NanA and α-NanBC mAbs and infected with strains EF3030 (*nanA/B*) or Sp#5 (*nanA/B/C*). Neutralization of NanA slightly reduced desialylation of the pulmonary conducting zone and further reduced desialylation of the respiratory zone in mice infected with EF3030 for 24 h. In the Sp#5 infection model, targeting NanA alone did not affect desialylation 24 h after infection. Neutralization of NanB and/or NanC did not have detectable effects in either model. However, antagonizing all neuraminidases with the α-NanA and α-NanBC mAbs led to greatly reduced desialylation of the conducting and the respiratory zones with a more pronounced effect seen in the EF3030 model (Figure 6C, Figure S2). Infection with Sp#5 for 48 h showed similar results as seen in the EF3030 model at 24 h, however, a relatively greater effect of NanB and/or NanC was observed in the Sp#5 model. Desialylation of the respiratory zones was only detected if the conducting airways of the same lung were also affected.

These data show that pneumococcal neuraminidases greatly affect the lung glycan-structure in acute murine pneumonia models. NanB and/or NanC can potently desialylate the murine lung epithelium in the absence of NanA.

### Neutralization of pneumococcal neuraminidases during acute murine pneumonia does not affect survival, lung bacterial burden, or host inflammatory responses

Upon detecting the pronounced inhibitory effects of the α-neuraminidase mAbs on desialylation of the lung epithelium by *S. pneumoniae*, we tested their efficacy on survival, bacterial organ load and inflammatory responses in mice.

We found that animals pre-treated with α-NanA or α-NanBC or both mAbs 48 h before challenge with lethal doses of *S. pneumoniae* strains EF3030 or D39 did not show improved survival compared to control animals (Figure S3A). Bacterial burden in the lungs was also measured in control and mAb-treated animals at 24 h post-infection with *S. pneumoniae* strains EF3030 or Sp#5. Of note, these data were generated from the lungs of the same mice used to assess pulmonary desialylation (shown in Figure 6C), confirming that the mAbs reached the lung tissue and exerted their inhibitory effect on neuraminidase activity. Again, no beneficial effect was observed (Figure S3B). Since cellular desialylation has been implicated as a pro-inflammatory signal, we determined the pulmonary levels of key cytokines and chemokines (including TNF-α, IL-1β, IL-6, interferons, KC, MIPs, MCP-1 and CXCR3 ligands) in the same animals used for the determination of the bacterial lung burden and desialylation. Despite clear induction of inflammatory host responses by *S. pneumoniae*, no significant changes were seen in mice treated with α-neuraminidase mAbs (data not shown).

These data suggest that neuraminidases, although active *in vivo*, do not play a pivotal role in this murine model of acute pneumonia.

## Discussion

Pneumococcal neuraminidases have been implicated as virulence determinants, mainly based on experiments using gene deletion mutants of laboratory strains of *S. pneumoniae*, both *in vitro* and *in vivo*. Previous studies reported a substantially reduced bacterial burden in the lungs of mice infected intranasally with *nan*A and/or *nan*B gene deletion mutant strains and improved host survival [39,40,68]. NanA was shown to contribute to nasopharyngeal colonisation [41,43,68–70]. However, these findings contrast to several other publications, where no such effects were observed *in vivo* [28,55,71–73]. Apart from differences in the bacterial strains and challenge doses, the reliability of generating true isogenic mutant strains may be a concern, especially when using a bacterium like the pneumococcus that is notorious for its genomic instability.

Whether the human antibody response against pneumococcal neuraminidases is protective is unknown. Although serum α-NanA antibodies increase during the first two years of life, no association with risk of subsequent carriage or acute otitis media has been demonstrated [74]. It is possible that α-NanA antibodies induced by prior exposure to *S. pneumoniae* do not reach a concentration required for protection, or that α-NanB and/or α-NanC antibodies are also required to reduce infection.

We generated two monoclonal antibodies that neutralize NanA, and NanB and NanC with high efficiency. This enabled us to characterize the involvement of the neuraminidases in *S. pneumoniae* lung infection models without potential artefacts associated with gene deletion mutant strains. The α-NanA mAb used in this study (#21155) was carefully selected to inhibit the activity of all major variants of NanA. A recent study described three representative NanA sequences (Genbank KJ850445.1, KT893379.1, KT893380.1) with distinct biochemical properties and susceptibilities to inhibitors [75]. These correspond to the clusters 1, 2, and 3, respectively, in this study. Importantly, all variants are equally recognized by mAb #21155. The other α-neuraminidase mAb used in this study (3C6-H7) neutralizes both NanB and NanC with comparably high potency.

Recently, compounds have been identified that can impair pneumococcal neuraminidase activity *in vitro* based on enzymatic or functional inhibition assays [76,77]. Our study extends these findings by demonstrating antibody-mediated inhibition of the neuraminidases during infection of human lung tissue and in mouse pneumonia models.

Neuraminidase activity of different pneumococcal isolates can differ greatly [30], for various reasons, such as the presence of one to three different neuraminidase genes [22] and NanA mosaicism that can influence enzyme activity [75]. Regardless of the underlying molecular mechanism, our study provides evidence that neuraminidase activity across a range of different pneumococcal isolates can be antagonized by the combination of α-NanA and α-NanBC mAbs *in vitro* and *in vivo*.

NanA and NanB have been shown to be involved in pneumococcal adhesion to epithelial cells and colonization of the host [36–38,69]. In this study, we investigated the contribution of the three neuraminidases to the interaction with the host. We did this by determining bacterial numbers recovered from host cells and from the supernatant after infection. We demonstrate that the presence of recombinant NanA and NanB, but importantly also NanC, increased the number of bacteria recovered from A549 lung epithelial cells during *in vitro* infection with a neuraminidase-deficient *S. pneumoniae* strain. On the other hand, in the absence of neuraminidase function, we detected reduced interaction of three different *S. pneumoniae* strains with epithelial cells. In these models, bacterial growth in the supernatant was affected if longer infection times (≥24 h) were used, but not at 3 h post-infection. Therefore, the increased bacterial numbers recovered from A549 cells at the early stages of infection likely arose from direct effects of the neuraminidases on bacterial interaction with the host cells (such as unveiling receptors to facilitate bacterial binding). At later stages, these differences were at least partly mediated by growth rates. MAb-based targeting of neuraminidases presumably leads to limited availability of sialic acid which can result in reduced growth, as shown before [37].

To more closely mimic the human lung environment *in vitro*, we used a human multilayered bronchial/epithelial 3D tissue infection model. In line with the results obtained with A549 cells, mAb-based inhibition of either NanA or NanB/C during pneumococcal infection reduced bacterial numbers recovered from primary human lung tissues 24 h post infection. Simultaneous targeting of all neuraminidases greatly enhanced this effect and protected the epithelial glycan-structure. In this model, bacterial growth in the supernatant was not influenced by the activity of the neuraminidases, therefore differences in bacterial numbers recovered from lung tissue potentially originated from direct effects at the lung cell surface.

The observed differences in the number of recovered bacteria in the absence of neuraminidases was greater with A549 cells compared to the 3D tissue culture model (up to 3 *vs*. 1 log), which is likely explained by the different effect on growth. This might be due to different amounts of, or access to sialylated glycan-structures. The malignant nature of A549 cells is known to affect surface glycosylation. For example, sialic acid molecules cluster differently on the A549 cell surface compared to human bronchial epithelial cells [78]. Moreover, A549 cells are not multi-layered and do not produce mucus. This might eliminate an important source of sialic acid used for growth during infection [30]. However, in the absence of this major natural barrier the pneumococcus may more readily access underlying sugars and adhere to host cells.

In murine pneumonia models, we demonstrate that targeting of all neuraminidases is required to protect the terminal epithelial pulmonary glycan-structure. MAb-based inhibition of neuraminidase-mediated desialylation of the respiratory zones (such as the alveolar areas with resident pulmonary and immigrating immune cells) was more readily observed than inhibition of desialylation of the conducting zones (such as the main bronchi and terminal bronchioles). This likely depends on bacterial density and spread as bacteria first have to pass through the conducting zones to reach the more distal respiratory zones. To gain more insight into desialylation kinetics, we infected mice with a *S. pneumoniae* strain (Sp#5) that carries all three neuraminidase genes (*nanA/B/C*). In spite of the inability of Sp#5 to induce lethal pneumonia in mice and its almost complete clearance from the lungs after 48 h of infection, desialylation was still clearly detectable at this time point. This suggests that bacterial clearance can be faster (host mediated in our model or due to antibiotics) than re-sialylation of pulmonary surface glycans.

Despite the observed efficient inhibition of *S. pneumoniae*-induced desialylation of murine lung tissues, we could not detect increased survival or a reduction in bacterial burden in the lungs of infected animals. This suggests that preservation of the terminal lung epithelial glycosylation pattern does not improve host defences in this murine model of lethal pneumonia. In this model, intranasal instillation of considerably high minimally-lethal bacterial challenge doses was required for each strain. An inherent limitation of this model is that a major step in the natural progression towards pneumonia, namely nasopharyngeal colonisation followed by bacterial translocation into the lower airways, cannot be recapitulated. It has been previously suggested that once sufficient numbers of bacteria are present in the murine lung, the pneumococcus employs virulence mechanisms to survive and further invade distal sites that do not involve NanA [39]. Our study supports this notion and extends this observation to the other neuraminidases (NanB and/or NanC). The importance of neuraminidases in bacterial growth by acquisition of host sugars might greatly differ in a non-inflamed compared to an inflamed organ, as the latter contains substantially higher levels of free glucose [18], which renders *S. pneumoniae* less dependent on sialic acid. It is noteworthy that mouse strain-dependent differences in the sialic acid composition have been reported [79]. Importantly, human and mouse airways differ in their pulmonary sialic acid repertoire [80,81] and the pneumococcus has evolved to specifically sense and respond to human sialic composition [82]. Therefore, the role of neuraminidases in human pneumonia is potentially underestimated by extrapolating from mouse models.

NanA was reported to increase pulmonary levels of pro-inflammatory cytokines by desialylating Siglec receptors on cell surfaces, which disrupts inhibitory signalling [46]. Pre-treatment of mice with the α-neuraminidase mAbs, and the proven neutralization of the neuraminidases could not reduce lung inflammation based on the lack of effect on cytokine and chemokine levels. It is likely that in such a stringent model with high bacterial inocula, the absence of a colonization phase and the multilobar nature of infection, more direct mechanisms such as pneumolysin-mediated killing of host cells drive the pathogenesis of pneumococcal pneumonia. This is underlined by the findings that administration of α-pneumolysin mAbs [12] or induction of serum antibodies by vaccination with recombinant pneumolysin [11] protects mice from lethal pneumococcal pneumonia.

In summary, this study demonstrates that NanA, NanB and NanC can remove terminal sialic acids from human lung tissue and confer an advantage to *S. pneumoniae* during its interaction with human lung cells *in vitro*. Antibody-based targeting of neuraminidases can greatly reduce neuraminidase activity *in vitro* and can also protect the murine lung epithelium from desialylation during acute pneumonia *in vivo*. However, we could not provide evidence for a decisive role of the neuraminidases in the acute phase of severe pneumonia in the mouse model applied. Non-invasive animal models, such as upper airway colonization and transmission to the lower airways, as well as otitis media might be more relevant models to further study the roles of neuraminidases during *S. pneumoniae* infections.
