# Supplementary material for: Assessing the function of pneumococcal neuraminidases NanA, NanB and NanC in in vitro and in vivo lung infection models using monoclonal antibodies
Source: Virulence. 2018 Oct 5;9(1):1521–38. doi: 10.1080/21505594.2018.1520545 (PMC6177239; doi:10.1080/21505594.2018.1520545)
Supplement: Supplemental Material [file kvir-09-01-1520545-s001.docx]

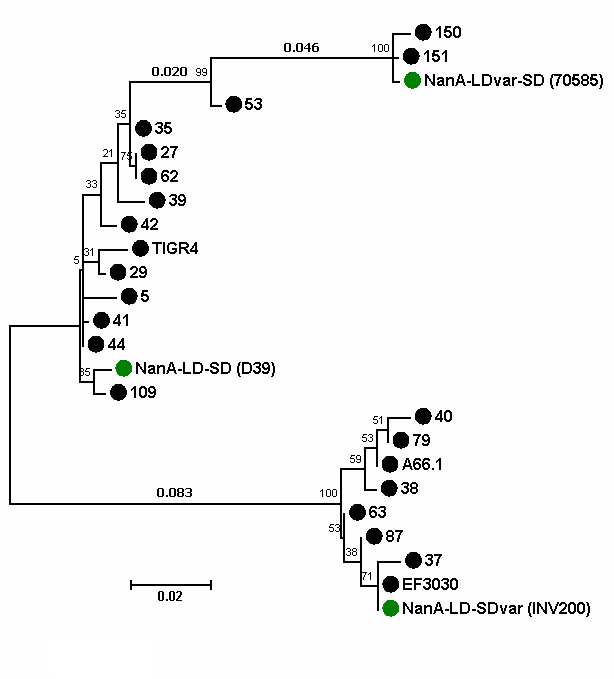


**Figure S1. NanA sequence diversity.** Evolutionary tree (generated with MEGA7.0.26 software, Maximum likelihood method, WAG model, Bootstrap n=500 replicates) of NanA (without the C-terminal-anchor) from *S. pneumoniae* strains (Sp#) used in this study (black), and NanA sequence variants (green). Numbers on branches indicate evolutionary distance (substitutions per site), Bootstrapping values are shown next to branch nodes.


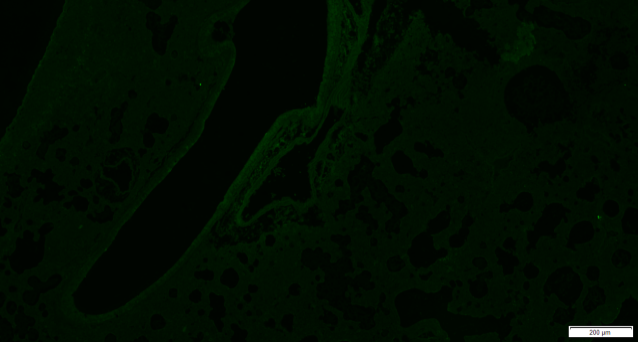

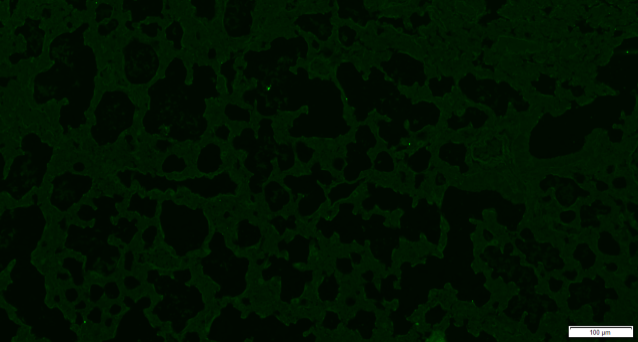

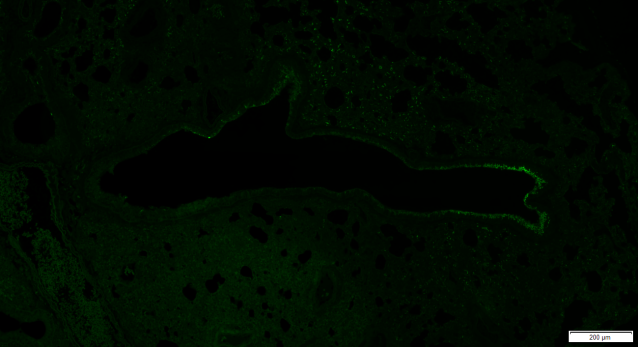

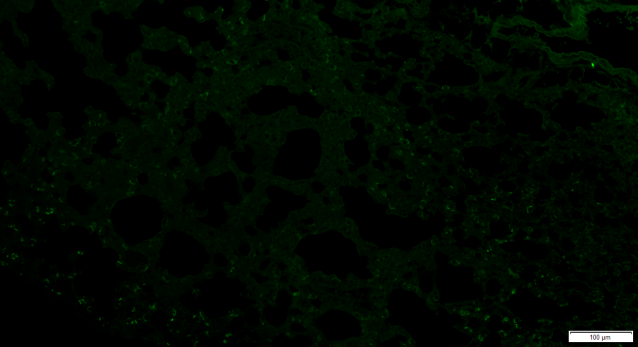

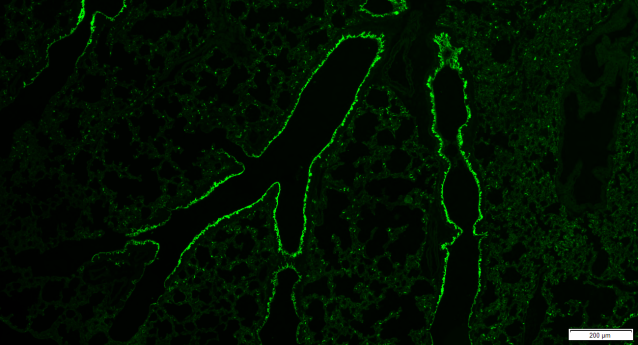

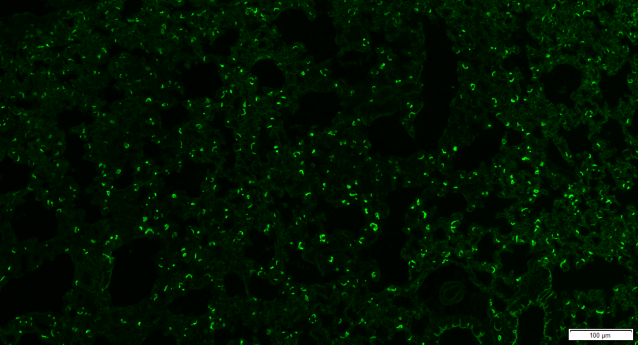


**0**

**1**

**2**

**Score**

**Respiratory zone**

**Conducting zone**

**Figure S2. Example images for semi-quantitative scoring of lung desialylation.** Mice (n=10) infected intranasally with *S. pneumoniae* in presence of α-NanA and α-NanBC or control mAb. Host sugar residues exposed by pneumococcal neuraminidases are stained with FITC-labeled peanut lectin (green). Scale bar= 100 μm. Desialylation scores were assigned as follows: 2 (desialylation), 1 (reduced desialylation), or 0 (no desialylation). Conducting zones (comprising main bronchi and terminal bronchioles) and respiratory zones (comprising respiratory bronchioles, alveolar areas and interstitial cells) were scored independently. Cumulative scores of ten mice per treatment (maximum score= 20, minimum score= 0) are summarized in Fig. 6C.

A

B

**Figure S3. Targeting neuraminidases during murine acute murine pneumonia does not ameliorate disease. A:** Survival of mice infected with *S. pneumoniae* EF3030 or D39 strains. **B:** Bacterial loads in lungs of mice infected with *S. pneumoniae* EF3030 or Sp#5 strains for 24 hours. Results are summarized as mean values relative to the control mAb treated groups +/- SEM. Statistical differences were evaluated via Mann-Whitney test, p values are indicated on the graphs.

**Table S1.** ***Streptococcus pneumoniae* strains used in this study.**

| **Human clinical *S. pneumoniae* isolates** | | | | | | |
| --- | --- | --- | --- | --- | --- | --- |
| **strain name** | **sample origin** | **serotype** | **PspA clade** | ***ply* allele** | ***nan* genotype** | **NanA cluster (Figure S1)** |
| Sp#5 | nose | 23F | 3 | 1 | *ABC* | 1 |
| Sp#27 | nose | 35 | 1 | 2 | *AB* | 1 |
| Sp#29 | trachea | 6A | not determined | 2 | *ABC* | 1 |
| Sp#35 | trachea | 11A | not determined | 2 | *ABC* | 1 |
| Sp#36 ^x^ | sputum | 15A | 5 | unclassified | *ABC* | mutant |
| Sp#37 | bronchus | 15B | not determined | 2 | *AB* | 2 |
| Sp#38 | BAL | 3 | 1 | 1 | *AB* | 2 |
| Sp#39 | nose | 7C | 5 | 2 | *AB* | 1 |
| Sp#40 | sputum | 18C | 5 | 2 | *AB* | 2 |
| Sp#41 | mini BAL | 1 | 1 | 5 | *AB* | 1 |
| Sp#42 | sputum | 4 | 4 | unclassified | *AB* | 1 |
| Sp#44 | nose | 42 | 1 | 2 | *AB* | 1 |
| Sp#53 | trachea | 19F | 4 | 15 | *AB* | 1/3 |
| Sp#62 | nose | 33F | 1 | 2 | *ABC* | 1 |
| Sp#63 | bronchus | 10A | 1 | 1 | *AB* | 2 |
| Sp#79 | sputum | 28F | 1 | unclassified | *AB* | 2 |
| Sp#87 | bronchus | 35B | 4 | 1 | *AB* | 2 |
| Sp#109 | bronchus | 14 | 1 | unclassified | *ABC* | 1 |
| Sp#150 | nose | 19A | 4 | 1 | *ABC* | 3 |
| Sp#151 | trachea | 6B | 6 | 1 | *A* | 3 |
|  | | | | | | |
| ***S. pneumoniae* protoype strains** | | | | | | |
| **strain name** | **sample origin** | **serotype** | **PspA clade** | ***ply* allele** | ***nan* genotype** | **NanA cluster (Figure S1)** |
| D39 | not recorded | 2 | 2 | 1 | *AB* | 1 |
| A66.1 | not recorded | 3 | 2 | 1 | *AB* | 2 |
| EF3030 | otitis media | 19F | 1 | 2 | *AB* | 2 |
| TIGR4 ^#^ | blood | 4 | 3 | 2 | *ABC* | 1 |
|  | | | | | | |
| ***S. pneumoniae* D39 gene deletion mutant strains** | | | | | | |
| **strain name** | **serotype** | **genotype** | **PspA clade** | ***ply* allele** | ***nan* genotype** |  |
| D39Δ*ply* | 2 | D39 ∆*ply::erm^R^* | 2 | 1 | *AB* |  |
| D39Δ*nanA* | 2 | D39 ∆*nanA::erm^R^* | 2 | 1 | *B* |  |
| D39Δ*nanB* | 2 | D39 ∆*nanB::kan^R^* | 2 | 1 | *A* |  |
| D39Δ*nanAB* | 2 | D39 ∆*nanA::erm^R^*  ∆*nanB::kan^R^* | 2 | 1 | ~~-~~ |  |

**^x^** Strain Sp#36 does not secrete functional NanA despite *nanA* being detected by PCR (frameshift mutation)

^#^ Strain TIGR4 secretes functional NanA (mutation in membrane anchor)

**Table S2. Oligonucleotide primers used for gene deletion mutant generation.** Lowercase DNA sequences denote primer sequences overlapping with the respective 5’ and 3’ sequences of the corresponding antibiotic resistance cassette.

| **Gene** | **Primer (5‘ - 3‘)** | | **Purpose of use** |
| --- | --- | --- | --- |
| *nanA* | *nanA*_KO1 | TTGATTTATTTCTTCACTTATTTGGGATTG | mutation |
|  | *nanA*_KO2 | ctgataaatatgaacatgatgagtgatcgtAGAAACCGCTCCTACCGATAGTT | gene deletion |
|  | *nanA*_KO3 | gacccggctaggctggcggggttgcGAGAAGAATTCTAAACATTTGATTTTGTAAAAAT | gene deletion |
|  | *nanA*_KO4 | TTTCATTCTCTAAATTAGTTTTGTTACCGT | gene deletion |
|  | *nanA*_*erm* insertion_CTRL_1 | GATTCTGATTGTGAGCTTGATTATATTTCC | deletion control |
|  | *nanA*_*erm* insertion_CTRL_2 | AGTCTTTTTCTAATTTGGAATTTAGTTTCA | deletion control |
|  | *erm*_1 | ACGATCACTCATCATGTTCATATTTATCAG | gene deletion/ deletion control |
|  | *erm*_2 | GCAACCCCGCCAGCCTAGCCGGGTC | gene deletion/ deletion control |
| *nanB* | *nanB*_KO1 | GCTGTATGACAATATAGAACGTCTCCGATA | gene deletion |
|  | *nanB*_KO2 | tacctagatttagatgtctaaaaagcAAACATGGTTAAATACGGTGTTGTTGGA | gene deletion |
|  | *nanB*_KO3 | tataacaagacgaactccaattcactgttTTTCCTTCTTTCGTCTACTTCACAGATC | gene deletion |
|  | *nanB*_KO4 | TTGTTAGCAATTCCCTATTCAATTGCC | gene deletion |
|  | *nanB*_*kan* insertion_CTRL_1 | GCGACAATCTTGATAAGAAAG | deletion control |
|  | NanB_*kan* insertion_CTRL_2 | TAGAATTGATGGTGCAAATAAATT | deletion control |
|  | *kan*_1 | AACAGTGAATTGGAGTTCGTCTTGTTATA | gene deletion/ deletion control |
|  | *kan*_2 | GCTTTTTAGACATCTAAATCTAGGTA | gene deletion/ deletion control |
| *ply* | *ply*_KO1 | ACTTTGGAAATTCTGAGTCAATCAGAAGACT | gene deletion |
|  | *ply*_KO2 | ctgataaatatgaacatgatgagtgatcgtCTTCTACCTCCTAATAAGTTCCTGGATTGA | gene deletion |
|  | *ply*_KO3 | gacccggctaggctggcggggttgcGAGAATGCTTGCGACAAAAAGAGGCGATGATCT | gene deletion |
|  | *ply*_KO4 | AGAAGAGTTTCTAAGCAAACCTCTAATACTCA | gene deletion |
|  | *ply*_mutag_CTRL_1 | GGATTATATTGTCAAGGTTATTGGCGACAAGCAT | deletion control |
|  | *ply*_mutag_CTRL_2 | GTCTACTCCAGACATATCATAGTTCAAGTAAATA | deletion control |

**Table S3. Oligonucleotide primers used for *nan*ABC gene screening and sequencing.**

| **Gene** | **Primer (5‘ - 3‘)** | | **Purpose of use** | **Source** |
| --- | --- | --- | --- | --- |
| *nanA* | Fw | ATAGACGTGCGCAAAATACAGAATCA | Screening/ sequencing | Pettigrew et al. (2006) |
|  | Rev | GTCGAACTCCAAGCCAATAACTCCT | screening | Pettigrew et al. (2006) |
| *nanB* | Fw | ACTACGAGGTGTTAATCGTGAAGG | screening | Pettigrew et al. (2006) |
|  | Rev | CCAATACCCGCAGGCATAACATC | screening | Pettigrew et al. (2006) |
| *nanC* | Fw | TGGGGTAAGTACAAACAAGAGG | screening | Pettigrew et al. (2006) |
|  | Rev | CTAATGGTACTGGCGAAAATCA | screening | Pettigrew et al. (2006) |
| *nanA* | 31R | TAACAGTACACCTTGACTGC | sequencing | Smith et al. (2013) |
|  | 31S | GTGTTCGATAAGGATTGAGC | sequencing | Smith et al. (2013) |
|  | A_seq_fw3 | TTGCGCACGTCTATTGTT | sequencing | this study |
|  | A_seq_rev3 | TGGGGTGATATCGGTATGG | sequencing | this study |
|  | A_seq_rev4 | AATGCTTCAAATGGTCAGAG | sequencing | this study |
|  | Sp_seq1 | GCATCGTATGGATAGCAG | sequencing | this study |
|  | Sp_seq2 | CTTGAACCAACACCATATC | sequencing | this study |
| *nanB* | B_seq_1 | TCATCGCTAGAAACCAAC | sequencing | this study |
|  | B_seq_2 | TCTTACCTGTAGTGGTTCT | sequencing | this study |
|  | B_seq_3 | GGCATAACATCAGCTAGTAAT | sequencing | this study |
|  | B_seq_4 | TGTATTTTCAACTGCCTG | sequencing | this study |
|  | B_seq_5 | TCGGAAGTTTCGTATATTG | sequencing | this study |
|  | B_seq_6 | AAACTAAAGAAGAATGGAGATA | sequencing | this study |
|  | B_seq_7 | GTGTTCTCTGAAACAGTTGA | sequencing | this study |
|  | B_seq_8 | GGGAGATATGATGGCAG | sequencing | this study |
| *nanC* | C_seq_1 | ACAAACATCTGATCCCAAC | sequencing | this study |
|  | C_seq_2 | ATGCTTGAATTACTCCTGG | sequencing | this study |
|  | C_seq_3 | AAAACT TACTCCAGCAGG | sequencing | this study |
|  | C_seq_4 | TTTGACTGAATCTGCTACTAC | sequencing | this study |
|  | C_seq_5 | CCCGGTCAACAAAATAGT | sequencing | this study |
|  | C_seq_6 | TAACATTGGCATTCGATG | sequencing | this study |
|  | C_seq_7 | GGAATCATTGAAAGCTCA | sequencing | this study |
|  | C_seq_8 | ACGTGTACCTTTTAGCGA | sequencing | this study |

**Table S3. Oligonucleotide primers used for *nan*ABC gene screening and sequencing.**

| **mAb** | **antigen** | **KD (M)** | **kon (M^-1^s^-1^)** | **koff (s^-1^)** |
| --- | --- | --- | --- | --- |
| **#21155** | NanA-LD-SD | 1.34E-09 | 1.49E+05 | 2.00E-04 |
|  | NanA-LDvar-SD | 3.96E-09 | 1.31E+05 | 5.19E-04 |
|  | NanA LD-SDvar | 2.42E-09 | 1.29E+05 | 3.11E-04 |
| **3C6-H7** | NanB | 5.86E-11 | 4.81E+05 | 2.82E-05 |
|  | NanC | 9.58E-10 | 1.95E+05 | 1.86E-04 |

**Table S4. Equilibrium and kinetic binding constants of α-neuraminidase mAbs to their target antigens.**
